# Gamification: a Novel Approach to Mental Health Promotion

**DOI:** 10.1007/s11920-023-01453-5

**Published:** 2023-10-06

**Authors:** Cecilia Cheng, Omid V. Ebrahimi

**Affiliations:** 1 Department of Psychology, The University of Hong Kong, Pokfulam, Hong Kong, China; 2 Department of Clinical Psychology, University of Oslo, Oslo, Norway; 3 Research Institute, Modum Bad Psychiatric Hospital, Vikersund, Norway

**Keywords:** Gameful experience, Gamified intervention, Mental wellness, Psychological symptoms, Psychotherapy, Serious play

## Abstract

**Purpose of Review:**

Gamification has emerged as a novel technique for improving mental health and enhancing treatment effectiveness. This paper provides an overview of gamification approaches to mental health intervention, identifies factors that may be related to variations in treatment effectiveness, and discusses possible strategies for tailoring gamified interventions to clients’ needs.

**Recent Findings:**

Recent research has documented the potential of gamified mental health interventions for bolstering mental wellness and mitigating psychological symptoms. However, their effectiveness may vary depending on study design-related factors and gender-specific considerations. Literature reviews have also identified yet-to-be resolved issues surrounding the possible strengths and weaknesses of the personalization versus standardization of gamification, as well as the potential benefits of gamification for increasing engagement versus the potential risks of over-engagement and behavioral addiction to gamified components.

**Summary:**

This review highlights the need for careful planning and execution of gamified mental health interventions to optimize their effectiveness and suitability for meeting clients’ individual needs and preferences.

## Introduction

Gamification, a technique that integrates gaming elements into non-game contexts, has gained popularity in promoting physical and mental well-being in recent years [1••, 2•]. Its origins date back to 1998, with the creation of the exergame *Dance Dance Revolution* for cardiovascular fitness and weight control [3]. Since then, gamification principles have been integrated into numerous games and mobile apps for both physical and mental health enhancement, such as *Nike* + for physical activity tracking and *SuperBetter* for achieving personal health objectives [4, 5]. Gamification has also been applied to mental health interventions and has the potential to enhance clients’ engagement by incorporating various gaming elements (e.g., points, rewards, badges, challenges) into therapeutic settings [6–9].

## Gamification Approaches to Mental Health Interventions

Gamification has emerged as a novel approach for improving mental health interventions, with two major applications in current practice. One approach involves directly developing gamified mental health interventions to create novel and more engaging treatments, whereas the other involves integrating gamification elements into existing mental health interventions to enhance their efficacy.

### Gamification as a Primary Strategy for Intervention

In the cyber age, advanced digital technologies originally created for gaming and entertainment such as virtual reality (VR) and augmented reality (AR) have been repurposed as tools to bolster mental wellness [10–13]. One key distinction between AR- and VR-based interventions is that AR overlays virtual elements onto a person’s real-world environment, whereas VR creates a fully virtual environment [12]. These technologies offer distinct advantages [12]. Specifically, VR-based interventions allow for fully customizable environments with fewer real-world distractions, enabling clients to more fully immerse themselves in virtual spaces. In contrast, AR-based interventions integrate with the real world, allowing for greater mobility and flexibility.

Both VR and AR technologies have been shown to provide clients with highly customizable scenarios for practicing skills such as social training and phobia exposure therapy in either mixed or fully constructed environments [9, 14]. These gamified interventions can foster a sense of safety for clients [15•], and are cost-effective and widely accessible [11]. Although some promising results have been reported for the use of gaming-intended technologies in treating specific psychiatric conditions, particularly anxiety disorders and PTSD, with minimal side effects [16, 17], there are differences among the various types of technology-based gamified interventions. For instance, a recent review found that immersive VR interventions may offer greater benefits in treating PTSD symptoms among military service personnel than conventional VR interventions [17]. Hence, when interpreting the efficacy of technology-based gamified interventions in mental health enhancement, it is essential to recognize the issue of variability in the diverse types of interventions and appropriately match the type of intervention to the target client group.

### Gamification Elements as Supplements to Existing Interventions

Another approach to incorporating gamification into mental health interventions is to add gaming elements as supplementary components to existing programs. For instance, a team of scholars sought to improve the effectiveness of a computerized cognitive-behavioral intervention designed for altering cognitive biases in clients diagnosed with depression. Specifically, they endeavored to alleviate the tediousness of repetitive tasks in this intervention by incorporating six key gamification components: goals, challenges, feedback, rewards, progress, and fun [18]. Their findings showed that all of these novel components were well-received by clients, who reported that the games involved in the therapy sessions were fun to play and that the purpose and instructions were clear. These findings suggest that clients generally accept the incorporation of gamification into existing treatments for mitigating depressive symptoms.

Another study examined the use of gamification elements in unguided cognitive-behavioral therapy for adolescents diagnosed with depression [7]. These elements included playful quizzes about the therapeutic content, puzzles, and mini-games involving rewards. The study demonstrated non-inferiority to treatment as usual for depression and high retention rates with no increased risks to clients, but it is noteworthy that the dropout rates increased when the intervention was implemented in real-world settings. Adolescent clients reported a desire for the game format to be as competitive and engaging as commercial games, highlighting an important challenge for the gamification literature. The availability of high-quality entertainment games has raised clients’ expectations for gamification components in mental health interventions, especially among adolescents who frequently engage in leisure gaming [19]. In such cases, it may be useful for clinicians to manage clients’ heightened expectations and clearly communicate the therapeutic purpose of the gamified intervention (i.e., to support mental health rather than to simply provide entertainment) to help clients approach the gamified intervention with the appropriate mindset.

Gamification components are increasingly being used in new forms of mental health interventions, including ecological momentary interventions delivered through mHealth systems such as mobile apps [20•]. Ecological monetary interventions first build on repeated assessments, typically multiple times per day, of a user’s experiences, symptoms, moods, and other relevant factors within their daily life context [21]. The data obtained from these assessments can be used to provide real-time interventions tailored to the user’s specific context and experienced state [20•]. For example, a study redesigned an mHealth system by integrating gamification techniques and interactive features to increase engagement and adherence among children receiving brief cognitive-behavioral therapy for anxiety symptoms [22]. The results indicated that the new gamified system was used more frequently than the previous non-gamified version, and young clients found the gamified system highly acceptable, useful, and engaging. These findings suggest that integrating an mHealth gamification platform into anxiety treatment for children has the potential to increase their involvement in the treatment.

## Nuanced Analysis of Gamification Effectiveness Across Studies and Samples

Although gamification has shown promise as a technique for improving mental health interventions, a more nuanced analysis of its efficacy in improving psychological outcomes is needed. In particular, it is important to examine the variability in gamification effectiveness across studies and samples and to identify key gamification components and factors that play an influential role in treatment outcomes. Meta-analysis is a powerful tool that can provide valuable insights into these issues [23].

Of note, a recent meta-analytic investigation revealed that gamified interventions generally elicit small to moderate positive effects on an array of mental health indicators, particularly on subjective well-being and quality of life indices [24••]. However, the effects of gamification on the reduction of anxiety and depressive symptoms seem more complex. Specifically, gamified interventions targeting anxiety related to specific objects or situations (e.g., acrophobia, test anxiety) were found to yield moderate to large effects, whereas those targeting generalized or global anxiety yielded null or small effects. Moreover, the meta-analytic findings showed substantial differences among the included studies that could not be attributed solely to sampling or random errors. Moderation and subgroup analyses were performed to unravel these intricate findings. The results identified three key factors—intervention duration, duration of follow-up assessment, and the gender composition of the sample—that played a crucial role in the observed differences in the treatment effect sizes across studies.

### Study Design Variations

The effectiveness of gamified interventions in mitigating anxiety symptoms has been found to vary by the duration of the intervention. Specifically, interventions with shorter durations are more effective in relieving anxiety symptoms than those with longer durations. This finding may be due to the primary focus of brief or short-term psychological interventions on the specific issues faced by clients. These interventions enable therapists to target specific problems and symptoms and to help clients develop strategies and skills to address those issues [25, 26]. Furthermore, the short duration of these interventions allows clients to see their progress quickly, which may increase their motivation to sustain their focused effort and impede dropout. Shorter psychological interventions are also more feasible for healthcare systems, particularly with regard to increasing cost-effectiveness [27, 28].

The efficacy of gamified interventions in reducing anxiety symptoms is influenced not only by the intervention duration but also by the duration of follow-up assessment. Among the cluster of studies that have evaluated the efficacy of gamified interventions, those with longer follow-up assessment periods generally exhibited stronger desirable treatment effects. Such variations may be related to the time required for clients to integrate the targeted behavioral changes into their daily lives, which is particularly important for complex or long-term behavioral changes involved in the treatment of psychiatric symptoms. This is often the case for gamified VR exposure and other cognitive-behavioral therapies. For instance, some gamified VR exposure therapies use the behavioral therapy technique of systematic desensitization to treat certain phobias [29, 30••]. This technique involves gradually exposing the client to feared objects or fear-evoking situations in a controlled manner, allowing the client time to work on their anxiety gradually before proceeding to the next stage. Other gamified interventions incorporate cognitive-behavioral therapy principles such as cognitive restructuring [31•, 32•], which also requires considerable time for clients to identify and challenge their maladaptive thoughts and replace them with more adaptive and realistic thoughts [33]. Given that clients need time to consolidate and integrate the therapeutic changes derived from gamified interventions into their daily lives, longer (vs. shorter) follow-up assessment periods may be better for capturing such long-term cognitive or behavioral changes that occur in real life.

Although longer follow-up periods for outcome assessment may allow clients additional opportunities to reinforce targeted changes through ongoing gaming experiences, it is worth considering the potential confounding effects of maturation, which are changes that occur over time independent of the intervention’s effectiveness [34]. To comprehensively evaluate the long-term impact of gamified interventions, studies should include a longer duration of follow-up assessment and, more importantly, a waitlist or active control condition to rule out the alternative explanation that any observed treatment effects are simply due to the passage of time.

### Gender-Specific Considerations

Considerations specific to gender are important in evaluating the effectiveness of gamified interventions. A recent meta-analysis revealed that on average, gamified interventions were more effective in mitigating anxiety symptoms among male clients than among their female counterparts [24••]. This disparity may be attributable to various factors, including differences in the extent of gaming experience, visuospatial processing, motivation, and genre preferences between genders. Studies indicate that male gamers tend to engage in gameplay more frequently and display greater visuospatial processing efficiency during gaming than female gamers [35–37]. These differences may influence clients’ performance in gamified interventions for anxiety reduction, particularly those that require visuospatial processing for navigating virtual environments such as VR biofeedback games [9, 38, 39, 40••].

Gender differences in gaming motivation have also been found, with male gamers generally exhibiting greater motivation for competition and challenge than female gamers [41]. To enhance motivation and engagement in gamified interventions, game developers often implement reward systems such as game points, badges, medals, trophies, and leaderboards [41–43]. These systems primarily emphasize the recognition of accomplishments through competitive, tangible rewards [44•], which are typically more appealing to male gamers who tend to have a stronger drive for achievement and competitiveness [45, 46]. Hence, gamified interventions that use tangible reward systems to reinforce competitiveness may be effective in motivating male clients and providing them with a more engaging and rewarding experience. In contrast, female gamers tend to have different motivational needs and preferences, such as being more inclined toward cooperative gaming and games that emphasize social affiliations [45]. Hence, it is necessary to explore alternative approaches to gamification that align better with the motivations and preferences of both male and female clients.

Gender differences in game genre preferences may also impact clients’ engagement with and the effectiveness of gamified interventions for anxiety reduction. Research has shown that male gamers tend to prefer fast-paced, action-oriented games such as first-person shooter and sports games, whereas female gamers tend to prefer puzzle and social games [35]. However, both genders have relatively equal preferences for adventure games. In light of these findings, researchers and game developers may consider these gender-related preferences when designing gamified interventions. This could involve creating games that are equally favored by both genders (e.g., adventure games) or tailoring gamification components to specific demographic characteristics. Although the aforementioned studies have revealed gender differences, it is important to note that individual differences within each gender group may be larger than the differences between gender groups. Therefore, it is essential to take into account individual differences when designing and implementing gamified interventions. By creating a more inclusive and engaging experience that appeals to all clients, gamified interventions can be optimized to effectively bolster mental wellness in a broader group of clients.

## Personalized vs. Standardized Gamification in Intervention Design

Gamified interventions involve the use of gaming elements in therapeutic settings to motivate and engage clients to foster more positive treatment outcomes. However, as discussed above, clients may have varying needs, preferences, and backgrounds that affect their involvement and participation. Hence, gamification should not be regarded as a one-size-fits-all solution. Two major approaches have been proposed for designing and delivering gamified interventions: personalization and standardization. Both approaches have their advantages and disadvantages, and mental health practitioners must carefully consider their intervention’s goals, target population, and available resources when deciding between these approaches.

### Evaluating Personalized vs. Standardized Gamification

The personalized approach to gamification involves individualizing gamified interventions by tailoring them to the specific needs and preferences of clients, taking into account a range of factors such as their experienced problems, goals, personalities, and demographic characteristics [47••]. This approach facilitates the targeting of individual differences in cognitive abilities, learning styles, and preferences, as previously discussed. By integrating customized support and feedback, goal-setting, and game mechanics, the personalized approach can heighten the relevance of the gamified intervention to clients, thereby enhancing their adherence to the intervention and attaining more favorable treatment outcomes [48].

From the user’s perspective, studies have indicated a general preference for gamified apps that offer a greater degree of customization [49•]. Empirical evidence indicates that mobile apps that provide more extensive customization options are associated with improved engagement and sustained use compared with those with limited customization options [50•]. Some users have even expressed a desire for a greater degree of control over the extent of personalization, highlighting the concept of “personalized personalization” as a preferred option [51•]. This concept refers to the user’s ability to decide not only the app’s content but also the level of customization itself, enabling users to customize their gamified experience to meet their specific needs and preferences. In light of this, researchers and game designers can explore opportunities to accommodate the needs and preferences of clients who seek greater control over the degree of personalization in gamified interventions.

Despite the benefits associated with personalization in gamified interventions, there are also limitations to this approach. Specifically, over-personalization can restrict the generalizability of interventions across diverse populations and make it difficult to assess their effectiveness [52]. Moreover, personalization can lead to inconsistencies in intervention delivery, which may impact its effectiveness. Standardization is an alternative approach that can address these issues by minimizing variability in the delivery of interventions and reducing the risk of bias [53]. Treatment adherence is a critical criterion that should be taken into account when assessing the effectiveness of a gamified intervention [54]. By adhering to standardized gamified setup and protocols, therapists can minimize variability in intervention delivery and ensure the dependability and validity of the outcomes obtained [55]. Adopting a standardized approach to gamification thus enables comparison of the findings derived from identical gamification settings, which can facilitate the replication of studies in distinct therapeutic settings and with diverse populations [51•].

It is worth noting, however, that the standardized or one-size-fits-all approach may not be suitable for all clients and may increase the risk of mismatching clients with gamified components, thus reducing engagement and increasing dropout rates. While standardized approaches may enhance the quality of the evidence obtained, it is important to recognize that the optimal degree of personalization and standardization may vary across populations and therapeutic settings. Therefore, a flexible and adaptable approach to gamification that strikes a balance between personalization and standardization is worth considering in the development and evaluation of gamified interventions.

### Deciding on Personalized vs. Standardized Gamification

When considering whether to use personalized or standardized approaches, mental health practitioners can make an informed decision by conducting a cost–benefit analysis, which involves weighing the advantages and disadvantages of different intervention designs to determine which one offers the largest net benefit [56]. Personalization may incur costs such as the time and resources required to develop and implement a highly customized gamified intervention [57–59]. In addition, there may be considerable challenges in managing and comparing the gamified intervention across diverse clients and settings. However, the benefits of personalization may include increased relevance and acceptability to the target population, which can potentially lead to better engagement and more favorable treatment outcomes [54].

Conversely, standardization may entail costs such as potential issues related to the gamified intervention’s relevance and acceptability to a heterogeneous group of clients, as well as potential challenges in implementing the intervention consistently across clients and settings [60]. Nevertheless, the benefits of standardization may include increased efficiency in delivery, scalability, and ease of implementation, leading to a more extensive reach and greater potential impact on the client communities [60]. Therefore, researchers and practitioners should carefully consider the costs and benefits of personalization and standardization approaches when designing gamified interventions, taking into account the specific goals and characteristics of the target population as well as the available resources. Further research is necessary to determine the optimal balance between these approaches to gamified interventions and provide guidance for the formulation of effective gamification strategies.

## Optimizing Engagement in Gamified Interventions

Client engagement has been a longstanding concern in mental health interventions, as a lack of engagement can adversely impact treatment effectiveness [61, 62]. Gamification has often been used to address this engagement problem and decrease dropout rates in various contexts, including mental health interventions [2•, 63•]. The underlying principle of gamification is to leverage gaming elements and mechanics to motivate clients to achieve their therapeutic goals and facilitate the therapeutic process by making it more enjoyable, engaging, and rewarding [24••, 64•].

### Enhancing Engagement Through Gamified Interventions

Various studies have demonstrated the potential benefits of gamification for improving retention rates in mental health interventions. For instance, *eQuoo* is a gamified app featuring a series of mini-games that can help clients strengthen a variety of skills, including emotion regulation and interpersonal communication [65]. Clients who used this app displayed lower attrition rates than those in both the control intervention group and the waitlist group without intervention [1••]. Similarly, the Mentalis Phönix app contains a set of gamified modules designed to teach skills for managing depressive symptoms [66]. The app presents psychoeducation materials in a game-design format, providing clients with a fun and engaging way to practice the skills they have learned. In an intervention evaluation study [31•], the app demonstrated a high retention rate of 93%, surpassing the retention rates observed in other gamified apps. Taken together, such findings provide some support for the efficacy of these gamified tools to help clients stay motivated and active during the intervention period.

Another body of research has reported that gamification can foster cognitive engagement, which refers to the extent to which clients are actively involved in the learning or problem-solving process and are mentally invested in the task at hand [67]. Gamification can enhance cognitive engagement in several ways. For example, gamification elements (e.g., trophies, leaderboards) can create a sense of challenge and excitement, which increases arousal and attention and in turn improves cognitive engagement. Furthermore, these elements can also promote positive emotions (e.g., enjoyment, curiosity), which have been shown to enhance learning and problem-solving processes by increasing cognitive skills such as cognitive flexibility, creativity, and problem-solving capabilities [68, 69].

### Balancing Engagement and Risks in Gamified Interventions

Despite the effectiveness of gamification in enhancing cognitive engagement, it may not necessarily facilitate other types of engagement such as behavioral engagement [70]. Specifically, gamification elements may increase behavioral engagement only for clients who find the gamified activities motivating and enjoyable. As previously mentioned, clients tend to have diverse gaming motivations and needs. Although gamification elements (e.g., badges, leaderboards) can create a sense of mastery as clients progress toward their goals, this gamification strategy may be more effective in fostering behavioral engagement for those with a stronger achievement motivation. Hence, different clients may require dissimilar types of incentives to become behaviorally engaged in gamified interventions.

Although client engagement is generally desirable for mental health interventions [71••], the potential issue of over-engagement also warrants scrutiny [72]. Gamification strategies intended to encourage engagement can become problematic when clients become excessively focused on specific gamification elements such as rewards and competition, leading to the neglect of other important aspects of their lives (e.g., interpersonal relations, job duties), potential disruption of their daily functioning, and the possible manifestation of gaming disorder symptoms (e.g., craving, tolerance) [73••, 74]. Hence, clinicians and game designers should be aware of the potential benefits and risks of gamified interventions and closely monitor clients for signs of over-engagement or other undesirable outcomes (e.g., gamification exhaustion, social overload) [73••, 75••].

Taken together, this paradoxical issue highlights the importance of achieving an optimal level of engagement that is critical for gamified interventions in mental health contexts. This optimal level may be attained if the level of engagement is sufficient to motivate clients to continue participating in the intervention and achieve the desired mental health goals, while avoiding harmful consequences such as over-engagement and behavioral addiction to gamification elements [75••, 76]. Mental health professionals should thus carefully balance the level of engagement when designing and implementing gamified interventions. They should strive to create a gamified environment that is motivating and engaging for their clients, without promoting over-engagement that can lead to undesirable consequences. This balance may be achieved by considering each client’s unique needs, motivations, and preferences, as well as by close monitoring for indications of both under- and over-engagement.

## Conclusions

By leveraging gaming elements and mechanics in mental health interventions, gamification has the potential to facilitate engagement and motivate clients to attain their therapeutic goals, which include promoting mental wellness and relieving psychological symptoms. While some studies have reported promising results with gamification as a standalone or additional component of existing mental health interventions, others have shown more modest benefits. Recent reviews of the literature suggest that these heterogeneities may be related to differences in the gaming elements used and the suitability of specific gamification components for clients with varying demographic characteristics. Therefore, the implementation of gamified interventions may require careful planning and execution to ensure their effectiveness and suitability for clients’ individual needs and preferences. Practitioners and game designers may consider weighing the potential benefits and costs of personalization versus standardization of gamification, particularly with regard to treatment adherence and the evaluation of effectiveness. By striving to strike a balance between a sufficient level of engagement and over-engagement, the design of gamified interventions could be optimized to help clients attain their mental health goals and improve their overall well-being. Table 1 summarizes all of these main points and recommendations for gamifying mental health interventions discussed in this article.

**Table 1:** Key recommendations for gamifying mental health interventions

Recommendation	Description
Tailoring gamification	
Recognize the contextual limitations of gamification as a universal solution	Tailor gamified interventions accordingly, recognizing the influence of clients’ varying needs, preferences, and backgrounds on their involvement and participation in order to optimize engagement and effectiveness
Achieve a flexible and adaptable approach through personalization and standardization considerations	Personalization involves tailoring interventions to individual clients’ specific needs and preferences, while standardization aims to minimize variability in delivery. Enhance effectiveness with a balanced approach that addresses unique intervention goals, target population characteristics, and resource limitations
Conduct a cost–benefit analysis	Evaluate the pros and cons of personalized and standardized approaches through a cost–benefit analysis, considering factors such as time, resources, relevance, acceptability, engagement, and treatment outcomes
Expand research effort to inform evidence-based strategies	Conduct additional research to identify the optimal balance between personalization and standardization in gamified interventions and to provide evidence-based guidance for more effective gamification strategies
Optimizing engagement	
Create a fun and pleasant therapeutic environment	Engagement is crucial for treatment adherence and effectiveness. Use gamified elements, modules, and apps to strengthen skills, provide psychoeducation, and make the therapeutic processes more enjoyable, engaging, and rewarding
Foster cognitive engagement	Use gamification elements and mechanics to incorporate challenge, excitement, positive emotions, and arousal, thereby improving cognitive engagement in learning and problem-solving processes
Consider diverse gaming motivations and needs	Customize gamification strategies to address the unique incentives necessary for behavioral engagement based on diverse gaming motivations and needs of refined subgroups of clients
Monitor for over-engagement and undesirable outcomes	Exercise caution regarding the potential risks associated with an excessive focus on gamification elements, as it may lead to problems such as neglecting other aspects of life and the emergence of gaming addiction symptoms. Vigilantly monitor clients for these signs of over-engagement and promptly adjust gamification components as needed

As research and practice regarding gamified mental health interventions are still in the nascent stages, further investigations and evaluations are needed to optimize their design and implementation. By exploring the potential of gamification to address specific mental health disorders and symptoms, as well as the factors that may influence its effectiveness, scholars and practitioners need to identify the optimal components and mechanics of gamified interventions for diverse populations and therapeutic contexts. Such research data would inform the development of evidence-based interventions that can improve the well-being of clients with mental health challenges. In addition, future research could explore the potential risks associated with gamification and identify approaches to circumvent them. In short, continued investigation in this emerging field is needed to guide the development of tailored gamified interventions that improve client engagement, treatment adherence, and mental wellness.
